# Polysaccharide from *Angelica sinensis* attenuates SNP-induced apoptosis in osteoarthritis chondrocytes by inducing autophagy via the ERK1/2 pathway

**DOI:** 10.1186/s13075-020-02409-3

**Published:** 2021-01-30

**Authors:** Chao Xu, Su Ni, Chao Zhuang, Chenkai Li, Gongyin Zhao, Shijie Jiang, Liangliang Wang, Ruixia Zhu, Andre J. van Wijnen, Yuji Wang

**Affiliations:** 1 Trauma Center, The Affiliated Changzhou No.2 People’s Hospital of Nanjing Medical University, 29 Xinglong Alley, Changzhou, 213003 China; 2 Medical Research Center, The Affiliated Changzhou No.2 People’s Hospital of Nanjing Medical University, 29 Xinglong Alley, Changzhou, 213003 China; 3 Department of Orthopedics, The Affiliated Changzhou No.2 People’s Hospital of Nanjing Medical University, 29 Xinglong Alley, Changzhou, 213003 China; 4 Department of Orthopedic Surgery and Biochemistry & Molecular Biology, Mayo Clinic, Rochester, MN USA; 5 Department of Orthopedics, The Third Affiliated Hospital of Gansu University of Chinese Medicine, 222 Silong Road, Baiyin, 730900 China

**Keywords:** *Angelica sinensis* polysaccharide, Autophagy, Chondrocytes, Osteoarthritis

## Abstract

**Objective:**

Chondrocyte apoptosis plays a vital role in osteoarthritis (OA) progression. *Angelica sinensis* polysaccharide (ASP), a traditional Chinese medicine, possesses anti-inflammatory and anti-apoptotic properties in chondrocytes. This study aimed to determine the protective role of ASP on sodium nitroprusside (SNP)-induced chondrocyte apoptosis, and explore the underlying mechanism.

**Method:**

Human primary chondrocytes isolated from the articular cartilage of OA patients were treated with SNP alone or in combination with different doses of ASP. Cell viability and apoptosis were assessed, and apoptosis-related proteins including Bcl-2 and Bax were detected. Autophagy levels were evaluated by light chain 3 (LC3) II immunofluorescence staining, mRFP-GFP-LC3 fluorescence localization, and western blot (LC3II, p62, Beclin-1, Atg5). Meanwhile, activation of the ERK 1/2 pathway was determined by western blot. The autophagy inhibitors, 3-methyladenine (3-MA), chloroquine (CQ), and a specific inhibitor of ERK1/2, SCH772984, were used to confirm the autophagic effect of ASP.

**Results:**

The results showed that SNP-induced chondrocyte apoptosis was significantly rescued by ASP, whereas ASP alone promoted chondrocyte proliferation. The anti-apoptotic effect of ASP was related to the enhanced autophagy and depended on the activation of the ERK1/2 pathway.

**Conclusion:**

ASP markedly rescued SNP-induced apoptosis by activating ERK1/2-dependent autophagy in chondrocytes, and it made ASP as a potential therapeutic supplementation for OA treatment.

**Supplementary Information:**

The online version contains supplementary material available at 10.1186/s13075-020-02409-3.

## Introduction

Osteoarthritis, a progressive and degenerative disease, is characterized by degeneration of the articular cartilage and osteophyte formation. Clinical manifestations include joint pain, swelling, joint deformity and, limited movement [1]. Many factors, including age, excessive weight bearing, oxidative stress, physiology, and biomechanical environment changes in joints, can result in OA [2]. Degeneration of the cartilage in OA is mainly due to the dramatically decreased self-repair ability of chondrocytes in a pathological status, presenting as low chondrocyte vitality, abnormally high apoptosis, and eventually loss of homeostasis of chondrocyte metabolism [3].

Many studies have reported that various factors could cause chondrocyte apoptosis, such as inflammation, oxidative stress, and mechanical stress [4, 5]. Growing evidence highlights that oxidative stress in chondrocytes is related to metabolic disorder and mitochondrial damage, which leads to massive apoptosis of chondrocytes. A previous study demonstrated that the nitrite levels, a stable end product of nitric oxide (NO) metabolism, are elevated in the serum and cartilage in OA samples. SNP, a NO donor compound, induces chondrocyte apoptosis via mitochondrial-dependent signaling [6].

*Angelica sinensis* polysaccharide (ASP), which is extracted with water as the initial extraction solvent, consists of xylose, galactose, glucose, arabinose, rhamnose, fructose, and glucuronic acid [7–9]. Some studies have reported that ASP exhibits gastrointestinal protective effects, immunomodulatory effects [10], antitumor activity [11, 12], and anti-inflammatory activity [13]. Furthermore, one study has shown the capacity of ASP to protect chondrocytes from H_2_O_2_-induced apoptosis via its antioxidant effects [14]. However, the influence of ASP on autophagy is unclear.

Autophagy, literally meaning “self-eating”, is an intracellular catabolic process of delivering cytosol and/or its specific contents to the lysosomes for degradation. The macromolecular constituents are then recycled and utilized by the cells [15]. Basal level autophagy plays an important role in cellular homeostasis through the elimination of damaged organelles and aggregated intracellular proteins [16]. On the other hand, during conditions of cellular stress, such as nutrient deprivation/starvation, hypoxia, pathogen infection, radiation, or anticancer drug treatment, the level of autophagy is augmented, resulting in adaptation and cell survival (cytoprotective response) [17]. Once this physiological augmentation of autophagy flux is genetically or chemically blocked, whether during the formation of autophagosome, the fusion of autophagosome and lysosome, or the degradation of autolysosome, cell death will generally increase under stress conditions [18, 19]. Cartilage degeneration and cell death caused by autophagy inhibition play a crucial role in the process of OA [20].

Additional evidence has shown that signaling pathway malfunctions in chondrocytes are involved in aging-related joint diseases such as OA. Extracellular signal-regulated kinase1/2 (ERK1/2) is related to chondrocyte apoptosis, as reported by Shakibaei et al. [21]. However, it was not clear whether there was a relationship between the ERK1/2 signal pathway and autophagy in chondrocytes after treating with ASP and SNP. Thus, our study aimed to identify if there is a link between ASP and autophagy on SNP-stimulated OA chondrocytes in vitro and which signal pathway is involved in it.

## Materials and methods

### Reagents

ASP was purchased from Shanghai Yilin Biotech. Co., Ltd. (Shanghai, China). The purity of ASP is approximately 92%. The component sugars are glucose, galactose, arabinose, rhamnose, mannose, and xylose. The average molecular weight of ASP is 85.0 kDa. ASP was dissolved in PBS and diluted with DMEM-F12 for the experiments. Collagenase II (Worthington Biochemical Corp., Lakewood, NJ, USA) was dissolved in DMEM at 2.5 mg/ml to digest the articular cartilage. Sodium nitroprusside (SNP) was purchased from Dandong Medical and Pharmaceutical Co., Ltd. (Heilongjiang, China), reconstituted in sterile normal saline at 40 mg/ml, and stored at 4 °C avoiding light. CQ, 3-MA, P276-00, and SCH772984 were purchased from Selleckchem (Houston, TX, USA).

### Isolation and culture of osteoarthritis articular chondrocyte

Cartilage tissue specimens were obtained from OA patients during joint replacement surgery in the Affiliated Changzhou No.2 People’s Hospital of Nanjing Medical University. All participants had signed a written informed consent prior to the subjects entering the study. In addition, the study was approved by the Nanjing Medical University Review Board. The information of subjects was performed in Table 1. All the tissues were carefully minced and digested with 2.5 mg/ml collagenase II in serum-free Dulbecco’s modified Eagle’s medium (DMEM) (Gibco BRL, Grand Island, NY, USA) for 4–6 h at 37 °C, filtered through a 70-μm cell strainer (BD, Durham, NC, USA), extensively washed with blank DMEM/F12, and finally cultured in DMEM/F12 supplemented with 10% fetal bovine serum (Gibco BRL, Grand Island, NY, USA), 50 μg/ml of ascorbic acid (AA, Sigma), 100 U penicillin, and 100 μg/ml streptomycin (which was referred as the full culture medium for chondrocytes) in a standard cell culture chamber containing 5% CO_2_. Non-adherent cells were removed after 3 days. Adherent cells were split at a ratio of 1:2 until 90% confluence. Chondrocytes were used from passages 2 to 3 in subsequent experiments.

**Table 1:** Characteristic of subjects investigated

Characteristics	OA
Total number of subjects	16
Age^a^, years	67.4
25th percentile	64
75th percentile	72
Number of female/male subjects	11/5
Disease duration^a^, years	5.7
25th percentile	3
75th percentile	8
ICRS grade^a^	2.8
25th percentile	2
75th percentile	3

^a^Median

### Determination of cell viability and proliferation by MTS assay

Cell viability and proliferation assays were performed using the tetrazolium compound-based CellTiter 96® AQueous One Solution Cell Proliferation (MTS) assay (Promega, Madison, WI, USA). OA chondrocytes were seeded at approximately 5000 cells per well in 96-well plates in triplicate for 7 days under regular growth conditions (DMEM/F12 with 10% FBS). After seeding for 24 h, ASP was added into the media, and then the MTS assay was performed daily according to the manufacturer’s instructions for the subsequent 6 days. Generally speaking, 20 μl of MTS solution reagent was pipetted into each well of the 96-well assay plate containing the chondrocytes in 100 μl of fresh culture medium. Then, the plate was incubated at 37 °C for 2 h in a humidified, 5% CO_2_ atmosphere, and the absorbance at 490 nm was recorded using an absorbance microplate reader (Elx808™ Bio-Tek Instruments, Winooski, VT).

### Edu cell proliferation assay

Chondrocytes were seeded in 24-well plates and treated with ASP and P276-00, and then cell proliferation rate was detected by the Yefluor 594 Edu Imaging Kits (Yeasen Co., Ltd., Shanghai, China) according to the manufacturer’s instructions.

### Cell apoptosis detection by DAPI staining

Chondrocytes were seeded on sterile glass slides coated with gelatin in a medium without AA and then treated with SNP alone or with ASP for an indicated time. Cells were fixed, and the nuclei were stained with DAPI (Sigma-Aldrich, MO, USA) in the dark for 5 min, and the fluorescence (Nikon Eclipse Ti, Japan) was observed.

### Detection of cell apoptosis rate by flow cytometry: Annexin V/PI staining

2 × 10^5^ chondrocytes were seeded in 6-well plates in a medium without AA. Cell apoptosis rates were detected by the Annexin V-FITC/PI kit (Vazyme Biotech Co., Ltd., Nanjing, China) according to the manufacturer’s instructions. Generally speaking, the cells were washed with ice-cold PBS and trypsinized. Removing the supernatant after centrifugation, the cells were resuspended in 100 μl binding buffer and incubated with 5 μl Annexin V-FITC for 10 min at room temperature avoiding direct light. Then, 5 μl PI and 400 μl binding buffer were mixed into the flow tube.

The apoptosis ratio was assessed with a flow cytometer (BD, Biosciences, San Jose, CA, USA), and the results were analyzed and assembled by the FlowJo software (Tree Star, Inc., USA).

### Immunofluorescence

2–5 × 10^4^ chondrocytes were seeded on sterile glass slides precoated with gelatin in a medium without AA. After the indicated treatment, cells were fixed in 4% paraformaldehyde at 4 °C for 15 min and blocked with PBS containing 5% normal goat serum and 0.3% Triton X-100 for 1 h at room temperature. Staining of the treated cells with LC3A/B (D3U4C) XP® Rabbit mAb (Alexa Fluor® 488 Conjugate, CST, USA) at 1/100 dilution was performed overnight at 4 °C in PBS containing 1% BSA and 0.3% Triton X-100. The nuclei were counterstained with DAPI in the dark for 5 min, and the fluorescence (Nikon Eclipse Ti, Japan) was observed.

### Western blot analysis

Cultured chondrocytes were lysed with RIPA buffer and boiled. SDS-polyacrylamide gel electrophoresis was conducted on a polyacrylamide gel and transferred to a polyvinylidene fluoride (PVDF) membrane. All antibodies, purchased from Cell Signaling Technology were used to detect the autophagy levels, apoptosis, proliferation, and signaling pathways. Rabbit anti-human β-actin polyclonal antibody was used to detect the actin signal as an internal control, and relative expression levels were quantified by running the Quantity One software. Antibodies information was performed in Table 2.

**Table 2:** Lists of antibodies

Antibodies	Manufacturer	Isotypes	Catalog
Atg5 antibody	CST	Rabbit IgG	#2775
Bax rabbit mAb	CST	Rabbit IgG	#5023
Beclin-1 rabbit mAb	CST	Rabbit IgG	#3495
Bcl-2 antibody	CST	Rabbit IgG	#4223
CyclinD1 rabbit mAb	CST	Rabbit IgG	#2978
p21 rabbit mAb	CST	Rabbit IgG	#2947
Phospho-p44/42MAPK(ERK1/2) rabbit mAb	CST	Rabbit IgG	#4377
P62 antibody	CST	Rabbit IgG	#5114
ERK1/2 rabbit mAb	CST	Rabbit IgG	#4695
Ras antibody	CST	Rabbit IgG	#3965
Raf antibody	CST	Rabbit IgG	#9422
p-MEK1/2 rabbit mAb	CST	Rabbit IgG	#9154
MEK1/2 mouse mAb	CST	Mouse IgG	#4694
LC3B antibody	CST	Rabbit IgG	#2775
β-Actin rabbit mAb	CST	Rabbit IgG	#4097

### mRFP-GFP-LC3 analysis

Chondrocytes were seeded in gelatin-precoated slides with a density of 5 × 10^4^ cells. One day after seeding, cells were infected with mRFP-GFP-LC3-labeled adenovirus (Genechem, Shanghai, China) according to the manufacturer’s instructions. The virus expresses the monomeric RFP-GFP-tagged LC3 (tfLC3) as an autophagic flux reporter comprised of LC3 protein fused with monomeric red fluorescent protein (mRFP) and green fluorescent protein (GFP). The GFP signal would be quenched within the lysosome lumen by the acidic and/or proteolytic environment. Yellow puncta which is consist of colocalized GFP (green) and mRFP (red) fluorescent signals in the cytoplasm indicate early autophagosomes, while the mRFP signals alone (red) represent late autolysosomes.

### Statistical analysis

Statistical analyses were performed using Prism (GraphPad Software, San Diego, CA, US). Unpaired Student’s *t* test was used for two groups and one-way ANOVA for more than two groups. The symbols *, **, ***, and ^#^ indicated *p* < 0.05, *p* < 0.01, *p* < 0.001, and *p* < 0.0001, respectively. All quoted *p* values were 2-tailed, and those less than 0.05 were considered statistically significant. All data are from *n* = 3 biological replicates.

## Results

### SNP dramatically attenuates chondrocyte viability whereas ASP rescues it

**Fig. 1:**
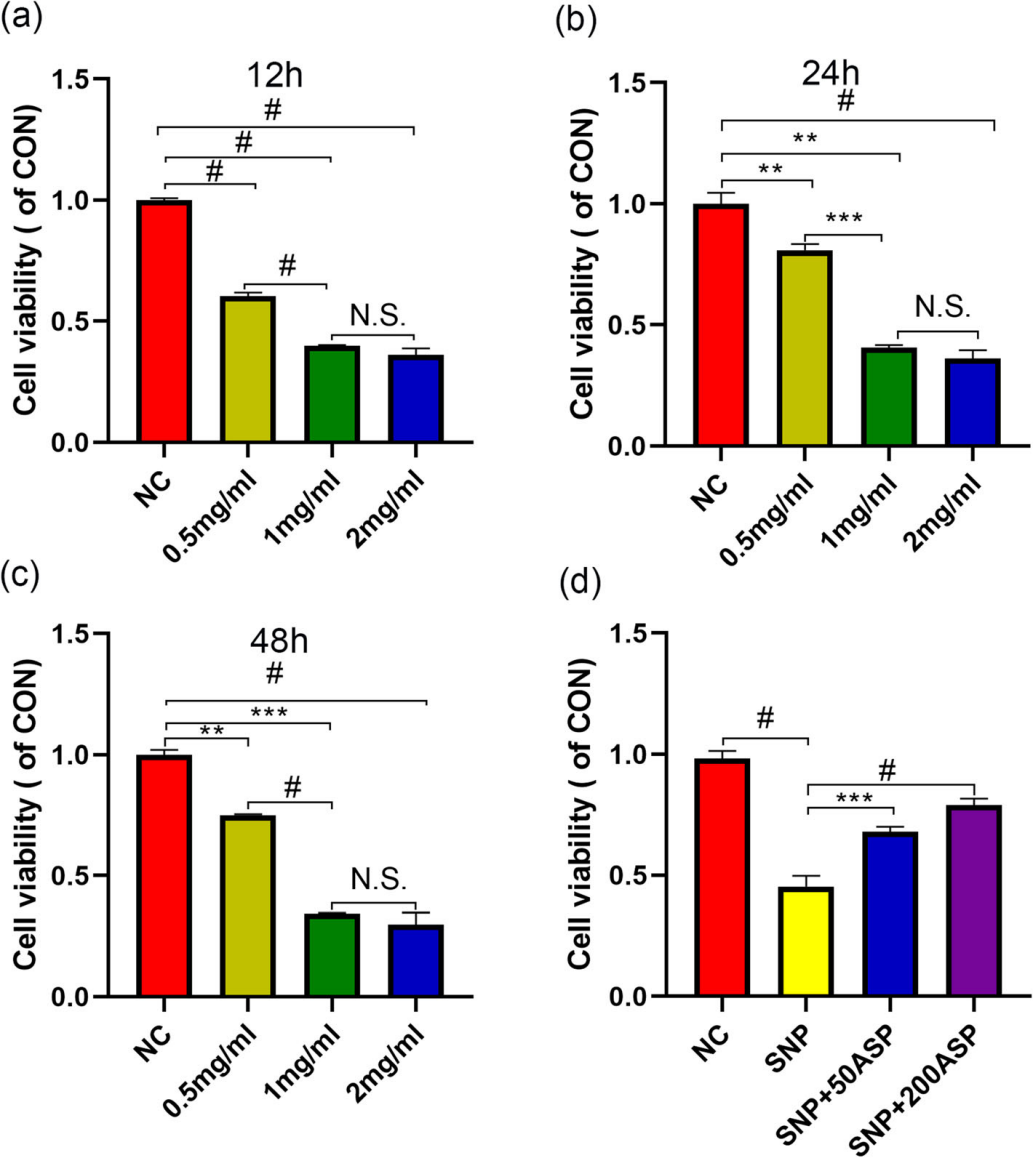
SNP treatment resulted in a reduction of chondrocyte viability compared with the control group in a dose-dependent manner. **a**–**c** OA chondrocytes were cultured with various concentration of SNP (0.5 mg/ml, 1 mg/ml, or 2 mg/ml) for 12 h, 24 h, and 48 h. **d** OA chondrocytes were pretreated with different concentrations of ASP (50 μg/ml or 200 μg/ml), then incubated with SNP for 24 h. Cell viability was analyzed with MTS. The results were presented as the mean ± SD of three independent experiments (*n* = 3). **p < 0.01, ***p < 0.001, ^#^*p* < 0.0001, and statistical significance was determined by one-way ANOVA

In order to determine the best dosage and time period of SNP application, OA chondrocytes were incubated with three different concentrations of SNP (0.5, 1, 2 mg/ml) for three different times, and sterile normal saline was added as a control. As demonstrated in Fig. 1a–c, the cell viability of chondrocytes was decreased in a dose-dependent manner. Especially when chondrocytes were treated with 1 mg/ml SNP, cell viability was reduced by approximately 50% (*p* < 0.01) after 12 h. Statistically significant differences in cell viability(12 h, 24 h, and 48 h) were not observed between the 1 mg/ml and 2 mg/ml groups (*p* > 0.05) but existed between the 0.5 and 1 mg/ml groups (*p*< 0.001). Therefore, 1 mg/ml SNP was used to induce apoptosis for the following studies.

In order to evaluate the protective role of ASP, chondrocytes were pretreated with 50 μg/ml or 200 μg/ml ASP for 2 h before 24 h incubation with 1 mg/ml SNP. Both concentrations of ASP, 50 and 200 μg/ml, remarkably rescued SNP-induced damage (approximately 30%, *p* < 0.001) as Fig. 1d showed, which suggested that ASP may protect chondrocytes from SNP-induced apoptosis.

### ASP promotes chondrocyte proliferation in a p21 and CyclinD1-dependent manner

**Fig. 2:**
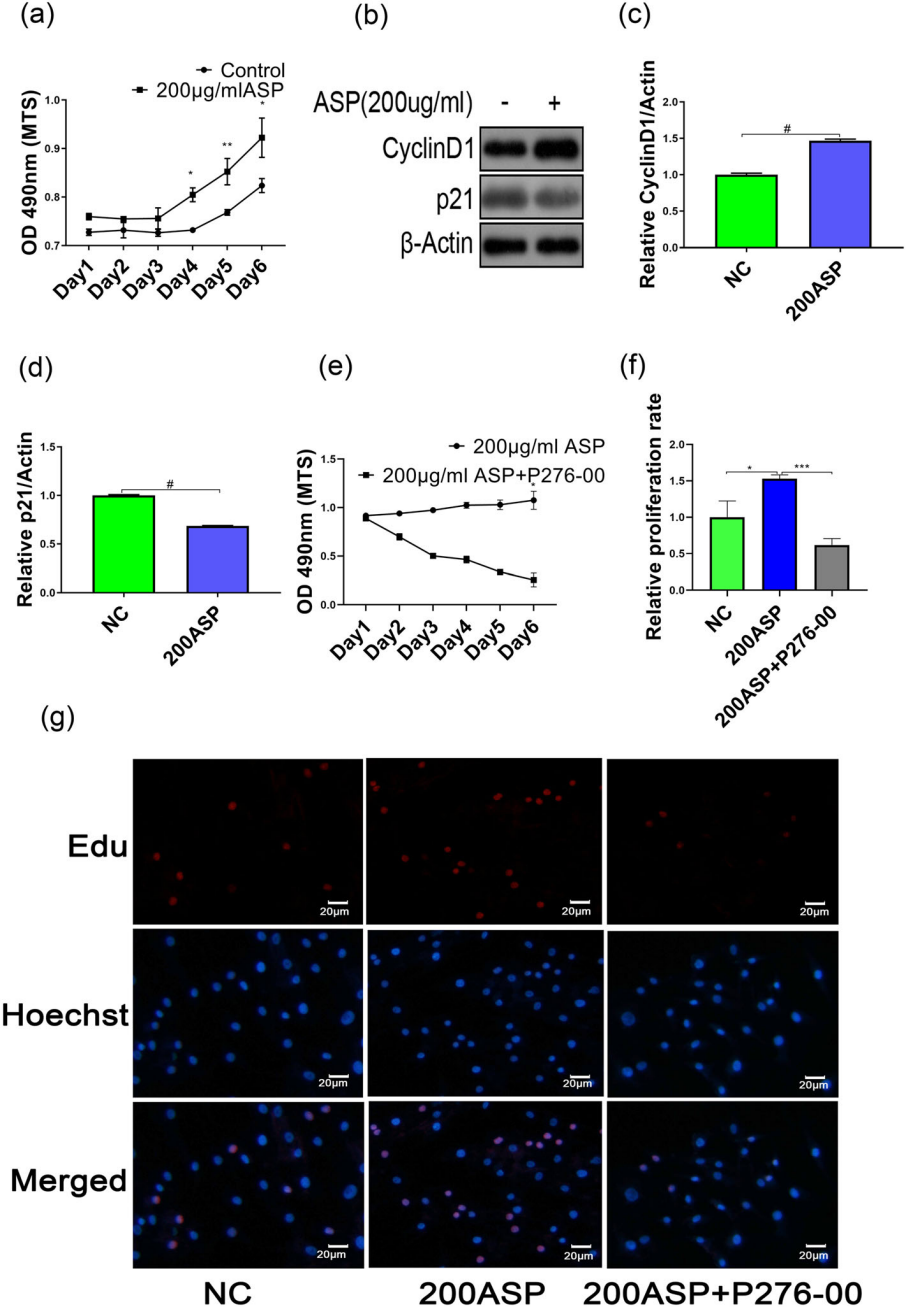
ASP-induced chondrocyte proliferation. **a** Chondrocytes were treated with or without 200 μg/ml ASP for 6 days. The results are presented as the mean ± SD of three independent experiments (*n* = 3). **b** Chondrocytes were collected after 4 days of exposure to 200 μg/ml ASP. Cyclin D1 and p21 were assessed by western blot (quantified in **c** and **d**). **e** After treatment with the culture medium, 200 μg/ml ASP alone or cocultured with the indicated concentration of P276-00 (2.5 μM) for 6 days. **g** Chondrocyte proliferation rate was detected by Edu assay at day 4 (quantified in **f**). Data were presented as the mean ± SD of three independent experiments(*n* = 3). *p < 0.05, **p < 0.01, ***p < 0.001, ^#^*p* < 0.0001, and statistical significance was determined by Student’s *t* test

To explore the effect of ASP on chondrocyte proliferation, we incubated chondrocytes with 200 μg/ml ASP for 6 days (ASP was added daily). The results showed that chondrocytes significantly increased from day 3 to day 6 compared to PBS control (Fig. 2a). To further examine if ASP-induced chondrocyte proliferation depends on the expression of p21 and cell cycle-related protein (CyclinD1), western blot analysis was conducted on day 4. As shown in Fig. 2b–d, ASP significantly decreased p21 (*p* < 0.0001) and increased CyclinD1 (*p* < 0.0001) protein levels. Additionally, P276-00, a CDK4/CyclinD1-specific inhibitor, was applied in combination with ASP for 6 days; the MTS results showed that ASP-induced chondrocyte proliferation was abolished when CyclinD1 was inhibited (Fig. 2e). Furthermore, we also used Edu assay to detect chondrocyte proliferation rate on day 4; the results showed the proliferation rate of chondrocytes in ASP-treated groups was increased by about 50% compared with the control groups. Meanwhile, P276-00 significantly inhibited the chondrocyte proliferation rate (Fig. 2f, g). Collectively, these data suggest that ASP enhances the proliferation of chondrocytes in a p21- and CyclinD1-dependent manner.

### ASP protects chondrocytes by inhibiting SNP-induced apoptosis

**Fig. 3:**
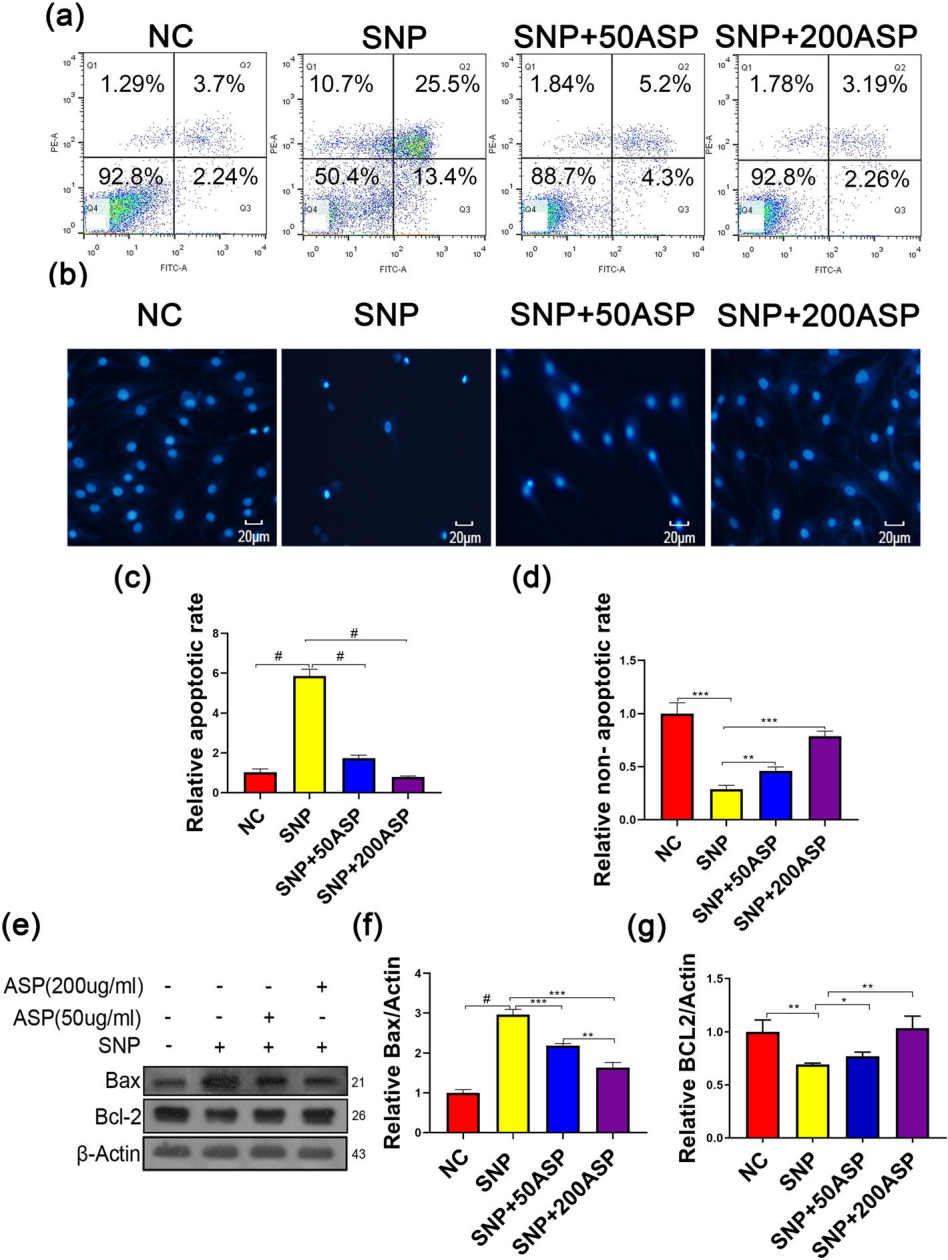
The protective effect of ASP on chondrocyte apoptosis. Chondrocytes were pretreated with ASP for 4 h then stimulated with SNP (1 mg/ml) for 24 h. **a**, **b** NC: chondrocytes were cultured in medium without AA for 24 h. SNP: chondrocytes were treated with 1 mg/ml SNP for 24 h. SNP+50ASP/SNP+200ASP: chondrocytes were pretreated with different concentrations of ASP (50 μg/ml, 00 μg/ml) for 4 h then incubated with SNP for 24 h. Chondrocyte apoptosis was detected by DAPI staining and flow cytometry assays (quantified in **c** and **d**). **e** The level of Bcl2 and Bax was measured by western blot (quantified in **f** and **g**). The results are presented as the means ± SD of three independent experiments (*n* = 3). *p < 0.05, **p < 0.01, ***p < 0.001, ^#^*p* < 0.0001, and statistical significance was determined by one-way ANOVA

To study whether the protective effect of ASP on SNP-induced cytotoxicity was mediated by the apoptotic process, we used DAPI staining and flow cytometry assays to assess chondrocyte apoptosis. Chondrocytes were pretreated with 50 μg/ml or 200 μg/ml ASP for 4 h and then treated with or without 1 mg/ml SNP for 24 h. For the negative control groups, the cells were treated with SNP only. As we expected, SNP significantly increased the percentage of apoptotic chondrocytes compared to the control groups; on the contrary, pretreatment with ASP significantly reduced the percentage of apoptotic chondrocytes (Fig. 3a, c). The DAPI staining results, which intuitively displayed the percentage of apoptotic cells, were consistent with flow cytometry assays (Fig. 3b, d). The balance of anti-apoptotic protein Bcl-2 and pro-apoptotic protein Bax plays an important role in the regulation of mitochondrial integrity and cell survival. To verify whether the mitochondrial-dependent apoptotic pathway was affected by ASP in chondrocytes, the expression levels of Bcl-2 and Bax were detected. Western blot analysis revealed that SNP significantly decreased Bcl-2 while increased Bax expression. On the contrary, ASP pretreatment significantly increased the expression of Bcl-2 while decreased the expression of Bax (Fig. 3e–g).

### ASP protects chondrocytes from apoptosis by inducing autophagy

**Fig. 4:**
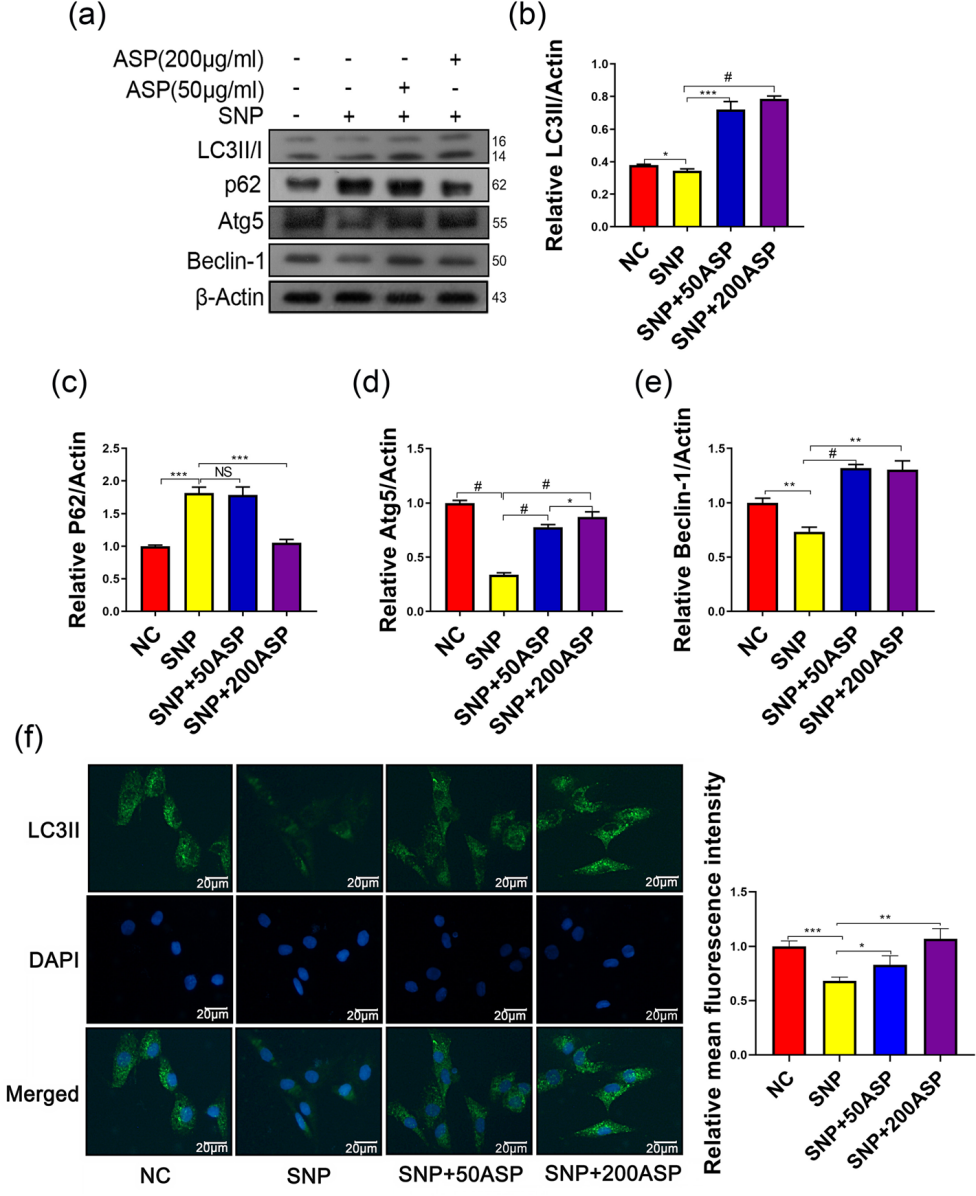
ASP-induced autophagy in chondrocytes stimulated with SNP (1 mg/ml). **a** Autophagy-related proteins (LC3II/I, p62, Beclin-1, Atg5) were detected by western blot. **b**–**e** The quantitative results are presented as the means ± SD of three independent experiments (*n* = 3). **f** LC3-II immunostaining. Significantly increased green bright puncta showed the formation of the autophagosomes. *p < 0.05, **p < 0.01, ***p < 0.001, ^#^*p* < 0.0001, and statistical significance was determined by one-way ANOVA

To determine whether the protective effect of ASP on chondrocytes was related to the autophagy activation, autophagic protein levels such as microtubule-associated protein light chain 3B (LC3II/I), p62, Atg5, and Beclin-1 were evaluated by western blot. The results showed that SNP significantly decreased Beclin-1, Atg5, and LC3II expression (*p* < 0.05). On the contrary, when pretreated with ASP, increased LC3II, Beclin-1, and Atg5 levels were found in chondrocytes (*p* < 0.05). Moreover, the expression of LC3II, Atg5, and p62 was regulated in a dose-dependent manner (Fig. 4a–e). LC3II immunofluorescence staining aligned with western blot results (Fig. 4f). Furthermore, SNP increased the expression of p62, whereas decreased levels of p62 were observable after ASP treatment, indicating ASP increased autophagolysosomal degradation (Fig. 4a, c). Together, these data suggest ASP-induced autophagy rescued SNP-induced apoptosis in chondrocytes.

### ERK1/2 signaling pathway is activated in ASP-induced autophagy

**Fig. 5:**
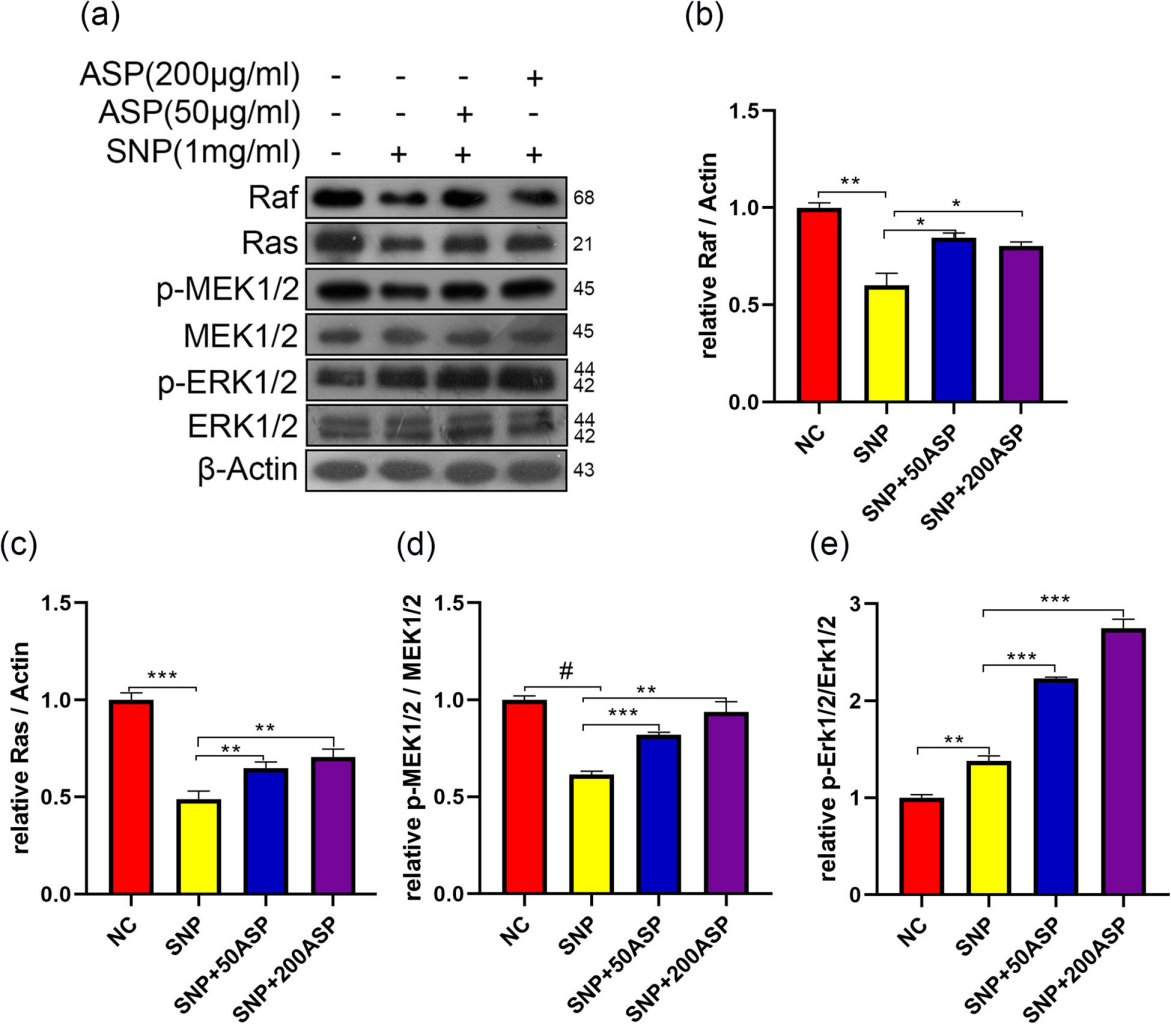
ASP activated the ERK1/2 signaling pathway in chondrocytes stimulated with SNP. Chondrocytes were pretreated with ASP for 4 h then stimulated with SNP (1 mg/ml) for 24 h. **a**, **b**. The level of Raf, Ras, p-MEK1/2, and p-ERK1/2 in chondrocytes after stimulation were measured by western blot. **b**–**e**. Quantitative analysis of Raf, Ras, p-MEK1/2, and p-ERK1/2 levels. The data are presented as the mean ± SD of three independent experiments (*n* = 3). *p < 0.05, **p < 0.01, ***p < 0.001, ^#^*p* < 0.0001, and statistical significance was determined by one-way ANOVA

**Fig. 6:**
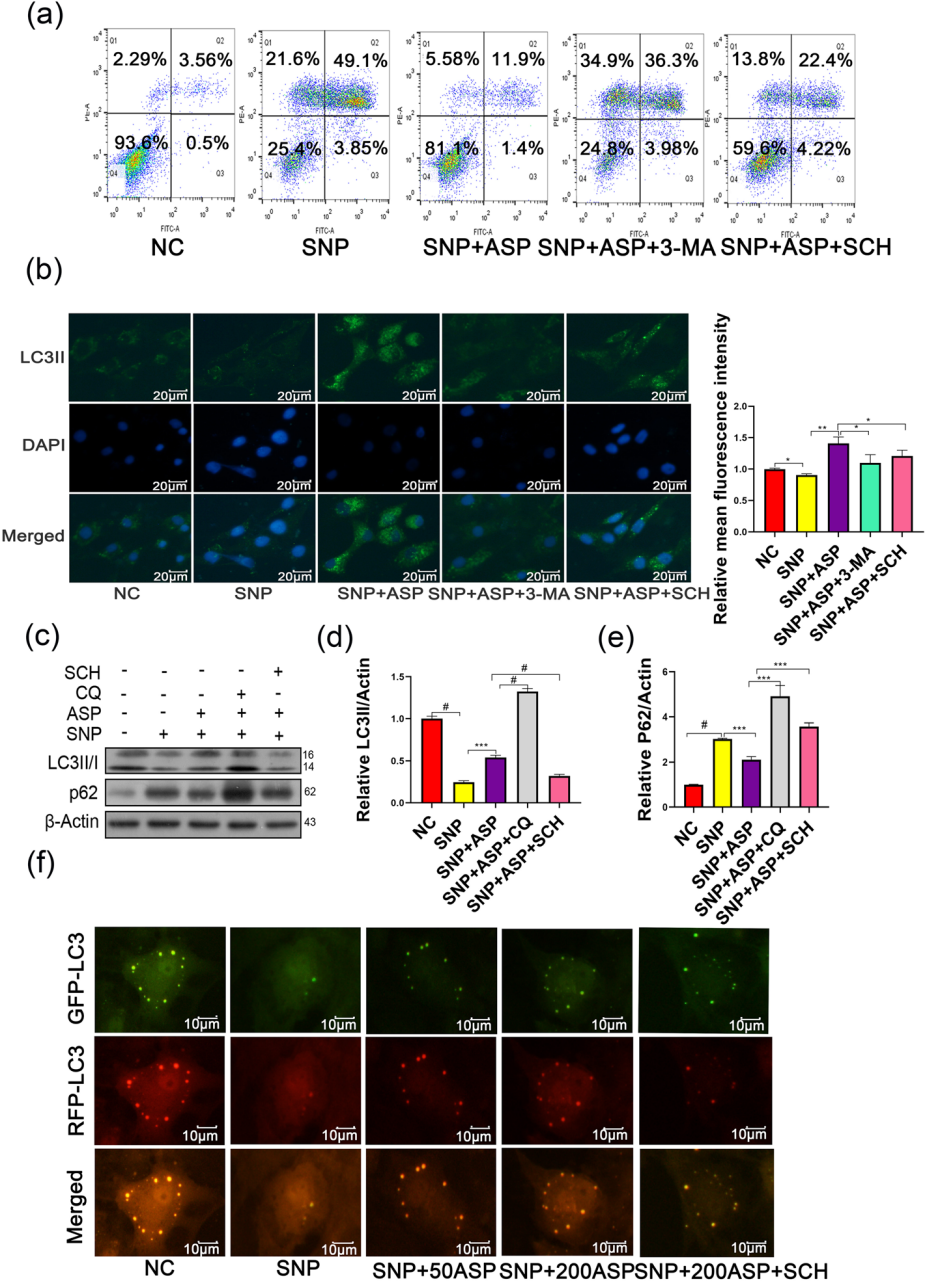
ASP diminished chondrocyte apoptosis via activation of autophagy. Chondrocytes were pretreated with 3-MA (10 μM), CQ (20 μM), and SCH772984 (10 nM) for 4 h before ASP treatment followed by SNP (1 mg/ml) for 24 h. **a** Chondrocyte apoptosis was measured by flow cytometry. **b** The formation of autophagosome was detected by LC3II immunostaining. **c** Levels of LC3II/I and p62 in chondrocytes after stimulation were measured by western blot. **d** Quantitative analysis of LC3II in chondrocytes. **e** Quantitative analysis of p62 in chondrocytes. **f** Representative microscopy images of tfLC3 puncta in chondrocytes stained for autophagosomes (green) and autolysosomes (red) as an indicator of autophagy flux. Autophagy increased in the ASP-treated groups in a dose-dependent manner. The data are presented as the mean ± SD of three independent experiments (*n* = 3). ***p < 0.001, ^#^*p* < 0.0001, and statistical significance was determined by one-way ANOVA

It is well reported that ERK1/2, one of the three main mitogen-activated protein kinase (MAPK) signaling pathways, regulates cell apoptosis and proliferation, and even affects autophagy. To illuminate the molecular mechanism of anti-apoptosis effect by ASP, we assessed the activation of the ERK1/2 signaling pathway in ASP-stimulated chondrocytes. As shown in Fig. 5a–e, ASP significantly increased the expression of Ras, Raf, phosphorylated MEK1/2 (p-MEK1/2), and phosphorylated ERK1/2 (p-ERK1/2) (*p* < 0.05), accompanied by increased expression of autophagy-related proteins LC3II and p62 degradation in SNP-treated cells, as shown in Fig. 6c–e. To further identify whether the ameliorated chondrocyte apoptosis was resulting from ASP-induced autophagy and ERK1/2 signal pathway activation, we used 20 μM CQ (autophagy flux inhibitor), 3-MA (autophagy inhibitor), and SCH772984 (selective ERK1/2 inhibitor) to pretreat chondrocytes. Flow cytometry results showed that the protective effect of ASP on chondrocyte apoptosis was significantly inhibited by 3-MA and SCH772984 treatment (Fig. 6a). In addition, ASP significantly increased bright LC3II puncta compared with the SNP group, while bright LC3II puncta were decreased by the inhibition of the ERK1/2 pathway and 3-MA treatment (Fig. 6b). To further evaluate the effect of ASP on autophagy flux, we used mRFP-GFP-LC3 autophagic puncta to visualize the level of autophagy. Enhanced autophagy was observed in the ASP-treated group whereas inhibited autophagy was detected with the ERK1/2 pathway inhibition (Fig. 6f). All these results suggested that ASP-mediated autophagy levels are linked to the ERK1/2 signaling pathway in SNP-stimulated chondrocytes.

## Discussion

In this study, we first demonstrated that ASP protects chondrocytes from SNP-induced apoptosis through the activation of autophagy. It has been reported that levels of nitrite, a stable end product of nitric oxide (NO) metabolism, are elevated in serum and synovial fluid samples of OA [22]. In addition, synovial cells and cartilage cells in OA produced large amounts of NO [23]. The negative effects of NO include enhancement of matrix metalloproteinase activity, a reduction in interleukin-1 receptor antagonist synthesis, and the promotion of apoptosis, which are closely associated with the occurrence and development of OA [24–26]. Thus, we chose SNP to induce NO-related apoptosis and discovered that chondrocyte viability declined in a time- and dose-dependent manner.

p53, which targets p21, can inhibit cell growth by blocking the cell cycle and induction of cell cycle arrest in the G0–G1 phase when the p53-p21 signaling pathway is activated [27]. One study has reported the role of p21 in potentiating cancer stem cells (CSC) via the activation of canonical Wnt signaling due to TCF1/Cyclin D1 upregulation. This results in the promotion of self-renewal and leads to the proliferation of CSC/progenitor cells that fuels tumor growth and metastasis [28]. Our study showed that ASP promotes chondrocyte proliferation via downregulation of p21 and upregulation of CyclinD1 expression, which were consistent with previous studies (Fig. 2).

Since SNP has been reported to induce mitochondrial apoptosis [29], alterations in mitochondrial membrane potential, and associated gene and protein expression levels, were investigated. Decreased mitochondrial membrane potential leads to increased membrane permeability, and mitochondrial membrane permeability may be regulated by the Bcl-2 family [30]. Conversely, the Bax protein increases the permeability of the mitochondrial membrane by forming activated oligomers, promoting Cyt-C release, and ultimately inducing apoptosis [31]. In our study, increased Bax expression and decreased Bcl-2 are involved in SNP-induced apoptosis, which was consistent with previous studies (Fig. 3).

Cetrullo et al. [32] demonstrated that oxidative stress inhibits the expression of autophagy-related proteins in chondrocytes and promotes apoptosis. Previous studies have shown OA cartilage produces a larger amount of NO compared with normal cartilage [33]. In addition, NO suppresses cartilage matrix synthesis and enhances degradation [34, 35]. Consequently, accumulating evidences showed that antioxidants, like *N*-acetyl cysteine (NAC) and eicosapentaenoic acid (EPA), can attenuate chondrocyte apoptosis caused by radical oxygen (ROS) and nitrogen (RNS) species in OA or after traumatic cartilage injury [36, 37]. Additionally, oxidative stress can lead to mitochondrial dysfunction, mitochondrial DNA damage, telomere instability, cell senescence, and anabolic dysfunction [38, 39]. As a result, more and more researchers are focusing on the therapeutic effect of antioxidants on OA. Polyphenols, like carnosol, hydroxytyrosol, curcumin, and genistein, have been revealed to have therapeutic potential in arthritis with regard to their antioxidant and anti-inflammatory features [40]. Another extensively investigated antioxidant is melatonin. It is reported to play a protective role in OA due to its ability to regulate apoptosis, ER stress, and mitochondrial activity [41].

Our study suggested that SNP, an oxidative stress inducer, inhibits autophagy levels, which was confirmed by Atg5, Beclin-1, LC3I/II, and p62 expression. LC3, an important constituent of autophagosomes, also plays an essential role in the fusion of autophagosomes with lysosomes for the degradation of damaged organelles by lysosomal enzymes [16]. LC3II has the ability to determine the membrane curvature, thus has a role in regulating the size of the autophagosome [42]. The expression level of LC3II could be affected both genetically by autophagy-related genes like Beclin-1 and SQSMT1/p62 or chemically by 3-MA and CQ [43–46]. Beclin-1 allows nucleation of the autophagosome and the conversion of LC3B-I to LC3B-II through lipidation by an ubiquitin-like system to form the autophagosome [43]. SQSMT1/p62 has a receptor function to recognize ubiquitinated proteins that need to be removed from the cytoplasm during autophagy; its amount is generally considered to inversely correlate with autophagic activity [44]. 3-MA inhibits autophagy by blocking autophagosome formation via the inhibition of class III PI3K, whereas CQ block the autophagic flux by decreasing the fusion process of autophagosome and lysosome [45, 46]. In our study, we first demonstrated ASP increased autophagy-related protein LC3II, Atg5, and Beclin-1 expression, indicating ASP promoted autophagy of chondrocyte. Additionally, we also detected the expression of p62, an autophagy substrate known to recruit ubiquitinated proteins and gets degraded as autophagic flux progress. The results suggest that ASP decreases p62 accumulation induced by SNP and CQ treatment further enhances p62 expression, indicating ASP not only enhanced the LC3II expression but also activated the autophagic flux. To further explore the correlation between ASP-induced autophagy and SNP-induced apoptosis, we used 3-MA as an autophagy inhibitor to block autophagy initiation. The results suggested that the protective effect of ASP against SNP-induced apoptosis was partly inhibited. All these findings confirm that ASP-induced autophagy plays an important role in preventing SNP-induced apoptosis in chondrocytes.

Autophagy is regulated by multiple signaling pathways in chondrocytes. Inhibition of the NF-κB pathway promotes the expression of Atg5, Atg7, and LC3II and activates autophagy [47]. Shi et al. showed that autophagy levels were significantly inhibited after activation of the p38 signaling pathway in osteoarthritis [48]. In addition, Li et al. reported that the ERK1/2 signaling pathway activation was involved in chondrocyte autophagy, which protected chondrocyte from apoptosis [49]. Moreover, pathways such as AMPK/mTOR [50], PI3K/AKT [51], and AKT/mTOR [52] were associated with autophagy in chondrocytes. We found that ASP-induced autophagy plays a critical role in the prevention of SNP-induced apoptosis via the ERK1/2 signaling pathway (Fig. 5). In keeping with the previous results, we discovered that ASP induced increased expression of p-ERK1/2 accompanying with a high expression of LC3II in chondrocytes treated with SNP and downregulated the expression of p62 simultaneously. For further proof, the application of SCH772984, an inhibitor of ERK1/2, significantly blocked the ASP-induced autophagy as it decreased the expression of LC3II and restored the SNP-induced expression of p62. Taken together, our results clearly demonstrated that the modulation of ERK1/2 plays a key role in the regulation of autophagy in chondrocytes treated with ASP and SNP.

Nonetheless, there are two major limitations in this study that could be addressed in future research. First, this study was based on the primary cultured chondrocytes which were thought to lose their characteristic in vivo in the synovium niche. The reality of chondrocyte apoptosis due to OA progression would be more complicated. Secondly, besides oral intake of ASP, whether there are more efficient ways to treat OA with ASP still needs to be looked into.

## Conclusion

ASP decreases SNP-induced cartilage damage and enhances chondrocyte proliferation in a CyclinD1- and p21-dependent manner. Besides, ASP activates autophagy to protect chondrocytes from apoptosis, via the ERK1/2 signal pathway. In addition, inhibitors of autophagy and the ERK1/2 pathway significantly abolishes the anti-apoptotic function of ASP against SNP. These findings indicate that ASP might be a promising natural compound for the treatment of OA.

## Supplementary Information

**Additional file 1:** SNP viability.

## Data Availability

The datasets used in the present study are available from the corresponding authors on reasonable request.

## Abbreviations

OA: Osteoarthritis
ASP: *Angelica sinensis* polysaccharide
SNP: Sodium nitroprusside
NO: Nitric oxide
AA: Ascorbic acid
LC3: Microtubule-associated protein 1 light chain 3
SQSTM1/p62: Sequestosome 1
Beclin-1: Autophagy-regulated protein
ERK1/2: Extracellular signal-regulated kinase1/2
SCH772984: Inhibitor of the ERK1/2 signal pathway
RIPA: Radioimmunoprecipitation assay buffer
DMEM: Dulbecco’s modified Eagle’s medium
PVDF: Polyvinylidene fluoride
CSC: Cancer stem cells
CyclinD1: Cell cycle regulatory proteins D1
MAPK: Mitogen-activated protein kinase
Raf: Raf kinase, effector of Ras
Cyt-c: Cytochrome c oxidase
Atg5: Autophagy-related 5
Atg7: Autophagy-related 7
Bax: BCL2-associated X protein
Bcl-2: BCL2 apoptosis regulator
3-MA: 3-Methyladenine
CQ: Chloroquine
mTOR: Mechanistic target of rapamycin kinase
AKT: Serine/threonine kinase
